# A Novel Technique for Drone Path Planning Based on a Neighborhood Dragonfly Algorithm

**DOI:** 10.3390/s25030863

**Published:** 2025-01-31

**Authors:** Sameer Agrawal, Bhumeshwar K. Patle, Sudarshan Sanap

**Affiliations:** Department of Mechanical Engineering, MIT Art, Design & Technology University, Pune 412201, India; sameer.agrawal2011@gmail.com (S.A.); sudarshan.sanap@mituniversity.edu.in (S.S.)

**Keywords:** path planning, dragonfly algorithm, aerial navigation, drone, AI technique, optimization

## Abstract

Autonomous aerial drone navigation is a rapidly growing topic of research due to its vast application in various indoor applications, including surveillance, search and rescue missions, and environmental monitoring. Current research focuses on the implementation of neighborhood dragonfly algorithms (NDAs) for path planning for single and multiple drones in various indoor environments containing stationary and moving obstacles. The collaborative behavior of dragonflies is a key concept in the current study that helps in exploring the solution space effectively and results in a faster convergence rate. To validate the performance of the proposed NDA approach, various environments are created in real time, and replicas of the same are generated using MATLAB software. Our analysis shows a close agreement between simulation and experimental results, with path length and navigational time differences of less than 5.7%. This underscores the consistency and feasibility of the NDA approach, placing the groundwork for robust and efficient drone navigation systems. The proposed NDA approach is also compared with those already developed, like IACO and PRM, in a similar environment. The NDA approach shows a better performance in terms of smooth path planning and path length optimization. The saving in path length is more than 5%.

## 1. Introduction

Drones, also known as unmanned aerial vehicles, have revolutionized a variety of fields. Drones have transformed the way tasks are carried out in diverse fields, including aerial photography and videography, surveillance, delivery services, mapping, and environmental monitoring. These nimble aircraft have extraordinary skills that allow them to operate in hazardous conditions and carry out missions that would be dangerous or impracticable for manned vehicles. Drones have developed into very complex machines with improved flight capabilities, enlarged cargo capacity, and extended operational ranges because of the quick development of technology. These drones should be able to fly autonomously and reach a defined target with obstacle avoidance without human intervention. Aerial navigation has terrific potential for research, as it has vast application in many fields like transportation logistics [1,2], health care [3], disaster management [4], the military [5], automated building inspection [6], marine inspection [7,8], scene reconstruction, aerial cinematography [9], and environmental exploration [10].

The selection of navigational techniques is one the most crucial stages in drone path planning, as it needs to travel in simple and complex environments. Pandey P. et al. [11] suggested that the 3D path planning process falls under the category of a hard optimization problem, where the objective is to find the best optimal solution among all the available solutions. The best approach for this would be the use of a non-deterministic algorithm that can provide a near-optimal solution. Broadly, 3D path planning strategies are divided into two major categories: the traditional approach and metaheuristic algorithms. The traditional approach works in a completely known environment where the environment map, position of obstacles, and goals are predefined for the robot. This approach divides the given environment into a set of nodes and then maps the nodes on the workspace configuration. Different types of traditional approaches in drone path planning are A-star (A*) [12], Dijkstra’s algorithm [13], D-star (D*) [14], rapidly exploring random trees (RRT), and RRT-star (RRT*) [15]. The major drawback of this type of algorithm is that it requires high computational time and inability to adjust due to the uncertainty present in the environment. To overcome this drawback, A. Gandomi et al. [16] suggested that a metaheuristic algorithm would be the best solution, as this would provide a problem-independent solution. These metaheuristic algorithms are fast and able to respond to the uncertainty present in the environment to find the optimal collision free path for the drone from start to goal. Some nature-inspired metaheuristic algorithms are evolutionary algorithm (EA), genetic algorithm (GA), differential evolution (DE), particle swarm optimization (PSO), ant colony optimization (ACO), artificial bee colony (ABC), simulated annealing (SA), tabu search (TS), improved bat algorithm, and cuckoo search (CS).

This paper presents the application of a dragonfly algorithm (DA) for the 3D path planning and obstacle avoidance of drones in the presence of static obstacles and moving obstacles. Many researchers have developed numerous applications based on DA [17,18,19,20]. For example, Jianjun et al. [21] proposed an improved DA for path planning for a heterogeneous multi-robot system. In this method, the 3D environment is modeled with neurons that are based on a cell grid system and bioinspired neural network. In a study conducted in 2018, Muthukumaran et al. [22] applied a dragonfly algorithm to facilitate path planning and static obstacle avoidance for mobile robots. They utilized diverse environments with static obstacles to generate simulation and experimental results. Ultimately, they concluded that the percentage difference between the simulation and experimental outcomes was remarkably low. Gururaghav et al. [23] used a DA for tracking the global maximum power point (GMPP) of a photovoltaic (PV) system. The exploration and exploitation characteristics of the DA were useful for efficient tracking of the GMPP. They also compared the performance of the newly developed DA with POS and a conventional tracking technique and found that the DA was superior in terms of time and energy consumption. A multi-objective DA (MODA) was used by Arun et al. [24] for optimal multi-response evolution of procedure parameters in creating reactions like surface hardness (H), surface roughness (Ra), and tool vibration displacement amplitude. Majdi et al. [25] proposed a wrapper-feature selection algorithm based on a binary DA (BDA), which is useful for reducing the number of features from the original feature set and improving the classification accuracy simultaneously. The results were compared with PSO and GA and found to be satisfactory in terms of classification accuracy. Abuomar et al. [26] used a BDA for swarm mobile robot cooperation by modifying two important behaviors: communication constraint and obstacle avoidance. Further, this BDA was modified by Abdelaziz I. et al. [27], who used different strategies for updating the five important coefficients used for feature selection. They proposed linear, quadratic, and sinusoidal BDAs, and after evaluation using 18 datasets, they found that the sinusoidal BDA outperformed all other proposed algorithms. Considering the importance of feature selection for many applications, Nagaraju et al. [28] modified the traditional DA by adding a convergence-and-fitness function to it and named it refined DA (RDA). The traditional DA algorithm has the drawback of falling into local optimal solutions, which decreases its performance in terms of feature selection and classification. To conquer this problem, Hongwei Chen et al. [29] proposed an optimized BDA algorithm based on Spark. This new method combined the global optimization ability of the dragonfly algorithm and the parallel computing ability of Spark, and significantly enhanced the functioning of the algorithm. Jingwei et. proposed a new hyper-learning binary dragonfly algorithm (HLBDA) that found the optimal subset of features for a given classification problem [30]. In the HLBDA, the hyper-learning approach helps the algorithm to escape from local optima and improve its searching behavior.

While the dragonfly algorithm on its own is highly effective in resolving optimization issues, it can also combine with other algorithms to tackle complex multi-objective optimization problems. In 2020, Muthukumaran et al. [31] combined the dragonfly and cuckoo search algorithms to find the best solution to the difficult multi-objective optimization problem of mobile robot navigation. The goals were to minimize path length, bending energy, and obstacle avoidance. They found that hybridized algorithms outperformed standalone algorithms in terms of generating short and smooth optimal paths and obstacle avoidance for mobile robot navigation, and hybridized algorithms outperformed standalone algorithms. Furthermore, they employed the hybrid dragonfly and cuckoo search algorithm (DA-CS) to create a sequential path for agricultural robots in a greenhouse setting. This hybrid DA-CS shows a better result as compared to PSO in terms of solution quality and computational efficiency. Kavitha et al. [32] proposed a combination of the modified dragonfly algorithm (MDA) and the whale optimization algorithm (WOA) named MDAWOA for the optimal scheduling of microgrids. An effective WOA approach modifies the dragonfly’s MDAWOA searching pattern.

Alejandro et al. [33] have suggested a new path planning technique based on the Q-learning approach. This approach can find the best path in the presence of a moving or static obstacle without any smoothing factor. The Q-learning approach also helped the algorithm to avoid falling into the local optimum. In [34], they combined particle swarm optimization (PSO) with the genetic algorithm (GA) to mitigate the issue of falling into the local optimum. Xiaobing et al. [35] hybridized the grey wolf optimizer (GWO) and differential evolution. This combination increases GWO utilization and is useful for solving the UAV path planning problem in dynamic conditions. In their study, Wang et al. [36] introduced a mutation mechanism into the traditional bat algorithm (BA), where mutations occur among the bats during the solution updating process. This mutation allows the UAV to identify a safe path that minimizes fuel consumption by connecting selected coordinate nodes while avoiding threats. This changed algorithm had a faster global convergence speed than population-based algorithms like differential evolution (DE), evolution strategies (ES), ant colony optimization (ACO), and particle swarm optimization (PSO). Abhishek et al. [37] integrated conventional particle swarm optimization (PSO) with the harmony search (HS) algorithm and the genetic algorithm (GA) separately. Compared to using GA or PSO alone, they found that this hybridized approach balances both exploitation and exploration in the search process, avoiding bias towards either aspect.

Out of all the algorithms discussed above, we chose the dragonfly algorithm (DA) for drone path planning because of its exceptional adaptability, efficacy, and resilience, which make it well-suited for optimization problems like drone path planning in indoor environments. DA provides a decentralized optimization approach, enabling individual agents to navigate complex environments while coordinating with their peers. Although DA has demonstrated an efficient performance in various optimization tasks, its application in multi-drone path planning remains largely unexplored in the literature. By utilizing DA’s unique capabilities in multi-drone contexts, we aim to introduce innovative solutions that use swarm intelligence principles to improve path planning efficiency, adaptability, and scalability indoors. This approach not only addresses the limitations of centralized algorithms but also facilitates collaborative decision-making and dynamic adaptation to environmental changes. Additionally, DA’s decentralized nature aligns well with the autonomous and distributed characteristics of drone swarms, making it an ideal choice for coordinating multiple drones in cluttered indoor spaces.

The dragonfly algorithm, with its basic concepts and proposed modifications in Section 2, structures this paper. The objective function formulation is in Section 3. Section 4 represents the simulation and experimental analysis results, with three sub-sections. The first and second sub-sections explain the simulation and experimental analysis, while the third sub-section shows the comparison between the simulation and experimental results. Section 5 represents the paper’s conclusion.

## 2. Dragonfly Algorithm (DA)

DA is a newly developed metaheuristic optimization technique proposed by Seyedali Mirjalili [38] in 2015. This algorithm is based on the swarm intelligence behavior of natural dragonflies. The remarkable characteristics displayed by static and dynamic swarming behaviors serve as the primary source of inspiration for the DA algorithm. Interestingly, these two swarming behaviors closely resemble the fundamental stages of optimization through meta-heuristics: exploration and exploitation. To elaborate, dragonflies, for instance, form sub-swarms and traverse diverse regions during the exploration phase, representing the objective of this stage. Conversely, in the exploitation phase, dragonflies operate within larger swarms and navigate in a unified direction, thereby capitalizing on favorable conditions within the static swarm.

We employ five fundamental primitive principles that dragonflies exhibit during their motion to simulate their swarm behavior. Here, we mathematically model these principles—separation, alignment, cohesion, attraction, and distraction. This paper reviews the existing DA using the calculus approach.

Let X be the position of the current individual, $X_{k}$ the position of the *k*th neighboring individual, N the number of neighboring individuals, and ∆X the step vector.

A. Separation corresponds to the static collision avoidance behavior observed in individuals, wherein they strive to prevent collisions with other nearby individuals. We can mathematically model this principle as follows:

(1) $$S_{i}=-\sum_{k=1}^{N}X-X_{k}$$

where $S_{i}$ represents the separation of the $i^{th}$ individual.

B. Alignment refers to the behavior in which an individual adjusts their velocity to match that of other nearby individuals within the same group. Mathematically, alignment is represented as follows:

(2) $$A_{i}=\frac{\sum_{k=1}^{N}V_{k}}{N}$$

where $A_{i}$ represents the alignment of the $i^{th}$ individual.

C. Cohesion represents the inclination of individuals to move towards the center of the swarm group. Mathematically, cohesion is defined as follows:

(3) $$C_{i}=\frac{\sum_{k=1}^{N}X_{k}}{N}-X$$

where $C_{i}$ represents the cohesion of the $i^{th}$ individual.

1. Attraction towards the food source is mathematically modeled by expressing the individual’s movement towards the food source as follows:

(4) $$F_{i}=X^{+}-X$$

where $F_{i}$ represents the food source of the $i^{th}$ individual and $X^{+}$ represents the position of the food source.

2. Distraction from enemies is mathematically modeled by incorporating the individual’s movement away from perceived threats or enemies. One can express the mathematical representation of distraction from enemies as follows:

(5) $$E_{i}=X^{-}+X$$

where $E_{i}$ represents the enemy of the $i^{th}$ individual and $X^{-}$ represents the enemy’s position.

The step vector which shows the direction of movement of dragonfly is,

(6) $${∆X}_{t+1}=\left(s.S_{i}+a.A_{i}+c.C_{i}+f.F_{i}+e.E_{i}\right)+w∆X_{t}$$

where s is the separation weight, a is the weight of alignment, c is the cohesion weight, f is the food function, e is the function of the enemy, w is the inertial weight, and t is the iteration.

In Equation (6), s, a, c, f, and e are different weights. By tuning these weights, different explorative (static swarm) and exploitative (dynamic swarm) behaviors can be achieved during optimization.

- In exploration, dragonflies have very low alignments while cohesion is high to attack prey, so we can assign a low alignment and a high cohesion weight.
- In exploitation, dragonflies tend to align their flying while maintaining proper separation and cohesion so we can assign a high alignment and a low cohesion weight.

The position vector of the $i^{th}$ dragonfly in *t* + 1 is calculated when surrounded by other dragonflies,

(7) $$X_{t+1}=X_{t}+∆X_{t+1}$$

The exploration is calculated by,

(8) $$Levy\;flight:L\left(x\right)=0.01\times \frac{r_{1}\times \sigma }{{\left|r_{2}\right|}^{\frac{1}{\beta }}}$$

where $r_{1},r_{2}\in \left[0,1\right],\;\beta$ is constant and σ is given as follows:

(9) $$\sigma ={\left(\frac{\left\lceil (1+\beta )\times \sin\left(\frac{\pi \beta }{2}\right)\right.}{\left\lceil \left(\frac{1+\beta }{2}\right)\times \beta \times 2\left(\frac{\beta -1}{2}\right)\right.}\right)}^{\frac{1}{\beta }}$$

where ⌈(x)=⌊(x−1).

The updated position of the dragonfly is as follows:

(10) $$X_{t+1}=X_{t}+L\left(d\right)X_{t}$$

where *d* is the dimension of the position vectors.

The fitness function is given by,

(11) $$F=w_{1}f_{1}+w_{2}f_{2}$$

where $w_{1}+w_{2}=1$.

The residual energy function in defined by,

(12) $$f_{1}=RE=\sum_{i=1}^{n}\left({IEL}_{i}-{CEL}_{i}\right)$$

where *RE* is the residual energy, *IEL* is the initial energy level, and *CEL* is the current energy level.

Hence, the function of the position of the drone is as follows:

(13) $$f_{2}=X_{t+1}\;\text{from}\;\text{Equation}\;(10)$$

Here is a summary of the dragonfly equation in calculus: Equations (1)–(13) are also multivariable calculus equations. Therefore, we refer to these equations as the real-valued function of the multiple real variables, i.e., $X,\;X_{k},\;V_{k},\;X^{+},X^{-}$, etc. The real-valued function of the *n* variable is represented by,

(14) $$z=f\left(x_{1},x_{2},\ldots .x_{n}\right);\;\left(x_{1},x_{2},\ldots .x_{n}\right)\in R^{n},\;z\in R$$

or the mapping is defined by,

(15) $$f:R^{n}\to R$$

Equation (14) is an explicit function, but it can also be defined as an implicit function as follows:

(16) $$\emptyset \left(z,\;x_{1},x_{2},\ldots .x_{n}\right)=0$$

This dragonfly equation is transformed into the calculus of the function of multiple variables by the following:

(17) $$S_{i}=f\left(X,X_{k}\right)$$

(18) $$A_{i}=f\left(V_{k}\right)$$

(19) $$C_{i}=f\left(X_{k},X\right)$$

(20) $$F_{i}=f\left(X^{+},X\right)$$

(21) $$E_{i}=f\left(X^{-},X\right)$$

(22) $${∆X}_{t+1}=f\left(s,a,c,f,e,S_{i},A_{i},C_{i},F_{i},E_{i},q,{∆X}_{t}\right)$$

(23) $${∆X}_{t+1}=t\left(X_{t},{∆X}_{t+1}\right)$$

(24) $$L\left(x\right)=f(r_{1},r_{2})$$

(25) $$X_{t+1}=f\left(X_{t},L\left(t\right)\right)$$

(26) $$F=f\left(w_{1}{,w_{2},f}_{1},f_{2}\right)$$

(27) $$f_{1}=RE=f({IEL}_{i},{\;CEL}_{i})$$

(28) $$f_{2}=f(X_{t+1})$$

**Figure 1:**
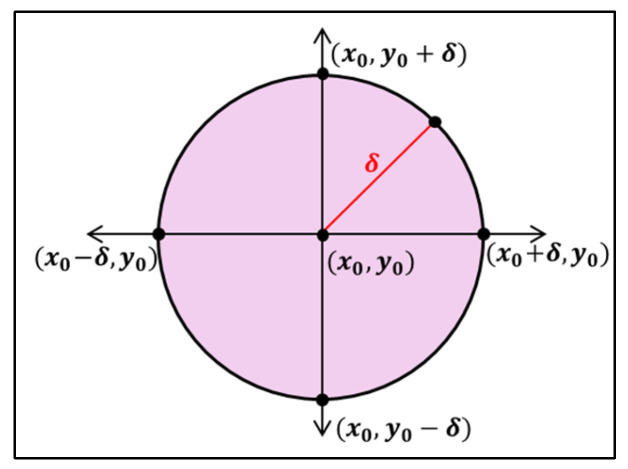
Neighborhood of a point $P\left(x_{0},y_{0}\right)$.

In the existing dragonfly system, there exists a set of finite neighbors and their respective positions. Calculus transforms these neighbors into points of the neighborhood. This is presented as follows:

Let $P\left(x_{0},y_{0}\right)$ be a point in $R^{2}$ for the function z=f(x,y). Then, the δ-neighborhood of the point $P\left(x_{0},y_{0}\right)$ is the set of all points (x,y) which lie inside a circle of radius δ with its center at the point $\left(x_{0},y_{0}\right)$. This is shown in Figure 1.

(29) $$\mathrm{Then},\;N_{\delta }\left(P\right)=\left\{\left(x,y\right):\sqrt{{\left(x-x_{1}\right)}^{2}+{\left(y-y_{1}\right)}^{2}}<\delta \right\}$$

$$\mathrm{since}\;\left|x-x_{0}\right|\leq \sqrt{{\left(x-x_{1}\right)}^{2}+{\left(y-y_{1}\right)}^{2}},\;\mathrm{and}\;\left|y-y_{0}\right|\leq \sqrt{{\left(x-x_{1}\right)}^{2}+{\left(y-y_{1}\right)}^{2}}$$

(30) $$\mathrm{Or}\;N_{\delta }\left(P\right)=\left\{\begin{array}{c} \left(x,y\right):\left|x-x_{0}\right|<\delta \;\\ and\;\left|y-y_{0}\right|<\delta \; \end{array}\right\}$$

There is a condition of the deleted δ-neighborhood of the point when the point $P\left(x_{0},y_{0}\right)$ is not included in the set. If the set of points meets the following criteria,

(31) $$0<\sqrt{{\left(x-x_{1}\right)}^{2}+{\left(y-y_{1}\right)}^{2}}<\delta$$

it is called the deleted neighborhood of $P\left(x_{0},y_{0}\right)$.

In general, the existing dragonfly equation is based on the $X_{k}-$ position or $k^{th}$ neighboring individual position. Since this position belongs to the set of real numbers, this is referred to as a real-valued function. The concept of the neighborhood of a point appears in the calculus of multiple-variable functions correspondingly. Basically, this is a mapping whose domain is a set of real numbers and whose codomain is also the set of real numbers. The range of the function is a subset of the real numbers. The performance of the dragonfly algorithm will be improved by the existence of two real positive finite numbers $\left(M,m\right)$ called the upper bound, if $\left|f(x,y)\right|\leq M$, and the lower bound, if $\left|f(x)\right|\geq M$, for all x,y∈ the subsets of real numbers.

The advantages of a neighborhood-based dragonfly equation are as follows:

1. There will be a broader domain of application with the neighborhood-based dragonfly equation.
2. Finding appropriate values for the dragonfly’s functions will be possible.
3. It will be possible to find increasing and decreasing dragonfly functions.
4. Finding the maximum and minimum values of the dragonfly function will be confirmed.
5. The dragonfly function’s series representation will be fixed.
6. Calculating errors in the dragonfly function will be set.
7. Reducing (σ) will be feasible.
8. Finding the mean values of the dragonfly function will be ensured.
9. A geometrical transformation of the dragonfly equation will be possible.
10. A combinatorial representation of the dragonfly functions will be achieved.

Therefore, the transformed neighborhood dragonfly equations of Equations (1)–(13) are as follows:

(32) $$N_{\delta }\left(S_{i}\right)=\sum_{k=1}^{N}X-N_{\delta }\left(X_{k}\right)$$

(33) $$N_{\delta }\left(A_{i}\right)=\sum_{k=1}^{N}N_{\delta }\left(V_{k}\right)$$

(34) $$N_{\delta }\left(C_{i}\right)=\frac{\sum_{k=1}^{N}N_{\delta }\left(X_{k}\right)}{N}-X$$

(35) $$N_{\delta }\left(F_{i}\right)=N_{\delta }\left(X^{+}\right)-X$$

(36) $$N_{\delta }\left(E\right)=N_{\delta }\left(X^{-}\right)-X$$

(37) $${N_{\delta }∆X}_{t+1}=\{s.N_{\delta }(S_{i})+a.N_{\delta }(A_{i})+c.{N_{\delta }(C}_{i})+f.N_{\delta }(F_{i})+e.N_{\delta }(E_{i})\}+wN_{\delta }(∆X_{t})$$

(38) $$N_{\delta }({∆X}_{t+1})=N_{\delta }(X_{t})+{N_{\delta }(∆X}_{t+1})$$

(39) $${N_{\delta }(∆X}_{t+1})={N_{\delta }(X}_{t})+L\left(d\right){N_{\delta }(X}_{t})$$

(40) $$N_{\delta }(F)={{w_{1}N}_{\delta }(f}_{1})+{{w_{2}N}_{\delta }(f}_{2})$$

(41) $$N_{\delta }(f_{1})=N_{\delta }(RE)=\sum_{i=1}^{n}N_{\delta }\left({IEL}_{i})-{N_{\delta }(CEL}_{i}\right)$$

(42) $$N_{\delta }\left(f_{2}\right)=N_{\delta }\left(X_{t+1}\right)\;\text{from}\;\text{Equation}\;(10)$$

Next, the neighborhood-based dragonfly algorithm is presented for real-time path planning. The movement rule of the drone is as follows. (This is the rule for updating the drone position.)

Front movement rule: $N_{\delta }\left(X_{t+1}\right)<X_{t+1}$;

Back movement rule: $N_{\delta }\left(X_{t+1}\right)>X_{t+1}$;

Left movement rule: $N_{\delta }\left(X_{t}\right)<X_{t}$;

Right movement rule: $N_{\delta }\left(X_{t}\right)>X_{t}$;

Diagonal movement rule: $N_{\delta }\left(X_{t+1}\right)<{N_{\delta }(X}_{t})$ or $N_{\delta }\left(X_{t+1}\right)>{N_{\delta }(X}_{t})$.

- Drone navigation rule for a static environment: The closed region concept is applied for the static navigation of drone. Basically, this is a bounded region together with its boundary. If there exists a real finite positive number M such that D can be enclosed with in a circle with the radius M and center at the origin. It is, $\left|OP\right|<M$, where OP is the distance of any point P in D from the origin.
- Drone navigation rule for a dynamic environment: There exists an open domain. It means a domain is open if for every point P in the domain D, δ > 0, such that all points in the δ-neighborhood of P are in D.

**Figure 2:**
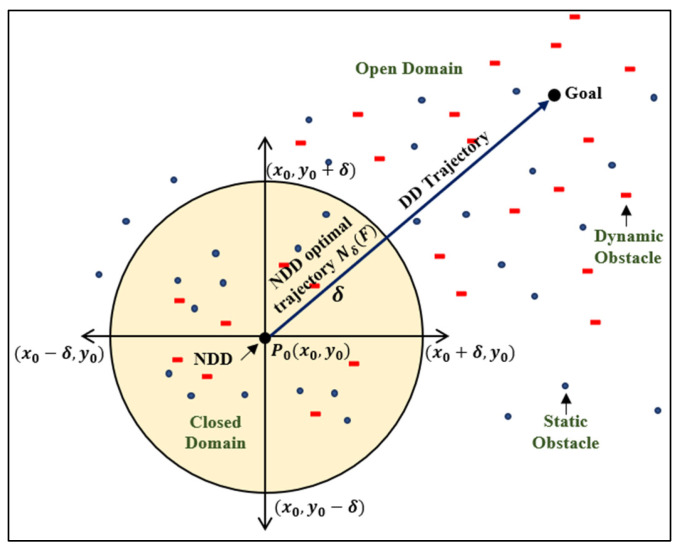
Neighborhood dragonfly drone (NDA) optimal navigation for real-time path planning in static and dynamic environments $\left(f:R^{2}\to R\right)\;\left(f:NDD\to G\right).$

**Figure 3:**
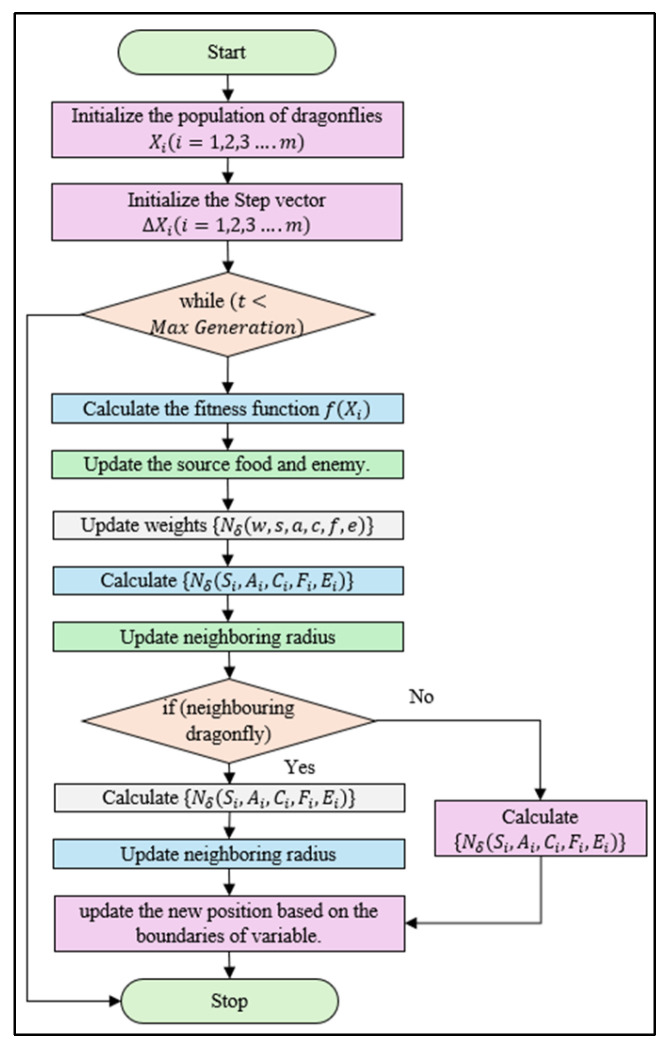
Structure of the proposed NDA approach.

**Figure 4:**
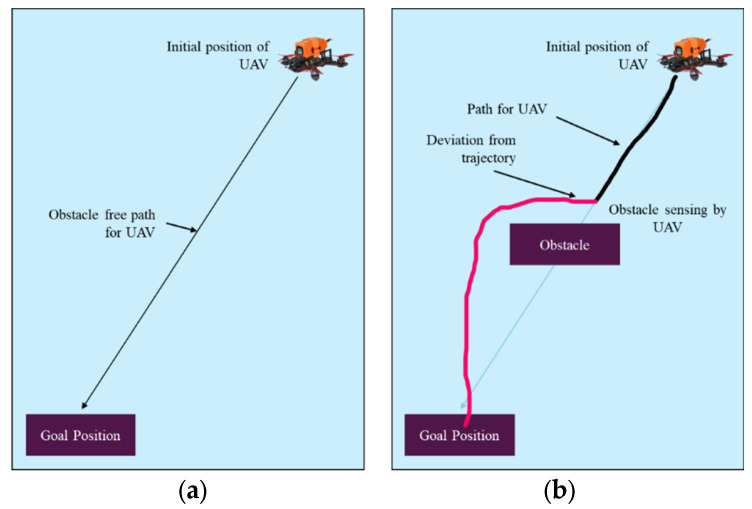
Motion of the drone. (**a**) Motion in an obstacle-free path. (**b**) Motion in a path with obstacles.

Figure 2 represents the δ-neighborhood of point p and the location of static and dynamic obstacles. The proposed NDA approach’s mechanism can be better understood from the pseudocode Algorithm 1 and flowchart Figure 3. When there is no obstacle present during motion, the drone will always follow the shortest path and direction. When the obstacle is present in the shortest path, the NDA will come into action, resulting in a deviation from the path as shown in Figure 4.
**Algorithm 1.** Pseudocode of the NDA algorithm.$\mathrm{Initialize}\;\mathrm{the}\;\mathrm{population}\;\mathrm{of}\;\mathrm{dragonflies}\;X_{i}(i=1,2,3\ldots .m)$$\mathrm{Initialize}\;\mathrm{the}\;\mathrm{step}\;\mathrm{vector}\;{∆X}_{i}(i=1,2,3\ldots .m)$$\text{while}\left(t<Max\;Generation\right)$ 
**do**
$\;\mathrm{Calculate}\;\mathrm{the}\;\mathrm{fitness}\;\mathrm{function}\;\mathrm{of}\;\mathrm{each}\;\mathrm{dragonfly}\;f(X_{i})$  Update the source food and enemy$\;\mathrm{Update}\;\mathrm{weights}\;\{N_{\delta }(w,s,a,c,f,e)\}$$\;\mathrm{Calculate}\;\{N_{\delta }(S_{i},A_{i},C_{i},F_{i},E_{i})\}$  Update neighboring radius  **if** (at least one neighboring dragonfly)$\;\;\;\mathrm{Update}\;\mathrm{the}\;\mathrm{velocity}\;\mathrm{vector}\;N_{\delta }\left(∆X_{t+1}\right)$$\;\;\;\mathrm{Update}\;\mathrm{the}\;\mathrm{position}\;\mathrm{vector}\;N_{\delta }\left(X_{t+1}\right)$
  **else**
$\;\;\;\mathrm{Update}\;\mathrm{the}\;\mathrm{position}\;\mathrm{vector}\;N_{\delta }\left(X_{t+1}\right)$
  **end**
  Update the new position based on the boundaries of the variable**end**

## 3. Methodology and Objective Function Formulation

The research methodology of this study was centered on the creation and assessment of the neighborhood dragonfly algorithm (NDA), aimed at improving UAV path planning in settings containing both static and dynamic obstacles. This approach involved a blend of simulation and experimental validation to thoroughly assess the algorithm’s effectiveness. Initially, we implemented NDA in MATLAB (R2019B) and tested it across various UAV scenarios, such as single UAV navigation among static obstacles and multiple UAVs operating in environments with both static and dynamic obstacles. We systematically varied key parameters like UAV speed, obstacle density, and simulation area dimensions to evaluate the algorithm’s adaptability and efficiency under varying conditions. To validate the NDA in real-world conditions, we developed UAVs in-house in a controlled indoor setting outfitted with sensors for precise obstacle detection, closely mirroring the simulated scenarios. The experimental design was intended to replicate the simulation scenarios to facilitate direct comparisons. We varied UAV speed, obstacle density, and operational area dimensions, establishing a robust framework for validation. We obtained comprehensive data on path length and travel time during both the simulation and experimental stages. The analysis revealed that the discrepancy between the simulated and experimental results is very small, affirming the NDA’s dependability and practical applicability. This minimal variance underscores the algorithm’s capability to perform effectively in real-world scenarios, demonstrating its potential for reliable UAV path planning in challenging environments.

Each dragonfly in the search area represents a possible solution. We can quickly find several optimal and suboptimal pathways by iterating through all the dragonflies in the search space. The capacity of metaheuristic algorithms to quickly offer many optimal and suboptimal solutions inside the search area is a major benefit. Consequently, the drone can redirect itself to a less-than-ideal option if it encounters an obstacle or another drone. We systematically varied key parameters such as drone speed, heading angle, obstacle density, and simulation area to evaluate the algorithm’s performance. Following this, experimental validation was conducted using UAVs in a controlled indoor setting equipped with LIDAR sensors for obstacle detection. The experimental setup was designed to replicate the simulated conditions for direct comparison. We carefully recorded and analyzed the data from both simulations and experiments. Path planning for a drone involves optimizing design parameters to achieve obstacle detection and avoidance, optimal path generation, fly-through trap-like situations, and so on. While traveling through the environment, the drones depend on sensors mounted on their embedded systems to gather essential information about their surroundings. This information helps it localize its position within unknown terrain. These sensors provide data on obstacles (position, size, shape) and other drones (position, size), enabling the drones to navigate towards their goal without collisions. The main aim of the current research work is to find an obstacle-free and optimal path for multiple drones in static and dynamic environments. Initially, we frame the path optimization problem as a minimization problem, formulate it into an objective function based on desired parameters like goal and obstacle positions, and then optimize it using the NDA approach.

The peripheral sensor of the drone provides the distance from obstacles and other drones as the first input parameter for the objective function selection parameter. In the NDA approach, the optimal position of the dragonfly within its group is determined by its proximity to the nearest obstacle and ensuring a safe distance from it. During each cycle, the position and step of all dragonflies are updated. Additionally, the neighborhood of each dragonfly is determined for updating the position X and step ∆X vectors by calculating the Euclidean distance between all dragonflies and selecting N of them. The dragonflies continuously adjust their positions until all obstacles are cleared. The distance between the best dragonfly and the nearest obstacle is given by Equation (43), and it is known as the Euclidean distance.

(43) $$D_{do}=\sqrt{{\left(x_{0}-x_{d1}\right)}^{2}+{\left(y_{0}-y_{d1}\right)}^{2}}$$

where $D_{do}$ is Euclidean distance between dragonfly and nearest obstacle, $x_{0},\;y_{0}\;$are the coordinates of obstacle in x and y direction and $x_{d1},\;y_{d1}$ are the coordinates of the dragonflies in x and y direction.

The location of nearby obstacles to drones is vital information for optimum path generation in complex environments. This can be calculated from Equation (44).

(44) $$D_{uo}=\sqrt{{\left(x_{0}-x_{u}\right)}^{2}+{\left(y_{0}-y_{u}\right)}^{2}}$$

$D_{uo}$ is the distance of drones from the nearby obstacle. $x_{0}\;$and$\;y_{0}$ are the x and y positions of obstacles, and $x_{u}\;$and $y_{u}$ are the coordinates of the drones.

From the group of random dragonflies, the optimum one is selected in such a way that it should have the maximum distance from the obstacle and the minimum distance from the goal. The distance from the goal $D_{ug}\;$can be calculated from Equation (45).

(45) $$D_{ug}=\sqrt{{\left(x_{g}-x_{d1}\right)}^{2}+{\left(y_{g}-y_{d1}\right)}^{2}}$$

where $x_{g}$ and $y_{g}$ are the position of the goal. The objective function for path planning optimization in the NDA approach is formulated by incorporating both obstacle avoidance behaviour and goal searching behaviour.

(46) $$f_{1}=K_{1}.\frac{1}{mino_{n}\in o_{s}|\left|D_{do}\right||}+K_{2}.\left|\left|D_{ug}\right|\right|$$

In Equation (46), $K_{1}$ is a fitting parameter that is responsible for path safety and smoothness and $K_{2}$ decides the maximum and minimum path length. For finding the optimum solution, the objective function (Equation (46)) is tested against the various parameters as shown in Table 1.

## 4. Simulation and Experimental Analysis

**Figure 5:**
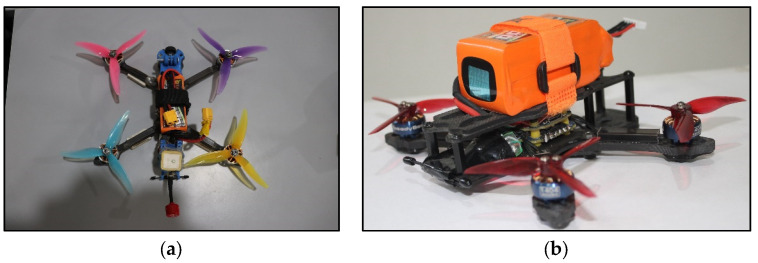
Inhouse built drone. (**a**) Carbon fiber frame 8-inch drone. (**b**) Carbon fiber frame 6-inch drone.

The focus of the current work is on path planning for drones in unfamiliar, static, and dynamic environments. We predefine the start and goal positions, but the drone must sense the obstacle’s location. The installed algorithm then guides it to avoid both static and dynamic obstacles and reach the goal. We develop a novel metaheuristic technique, the neighborhood dragonfly algorithm (NDA), to achieve this. We create various environments, ranging from simple to complex, containing static and dynamic obstacles as well as single and multiple drones, to evaluate the performance of the proposed NDA approach. MATLAB software (R2019) on a PC with an Intel (R) Core i5-8365U CPU at 1.60 GHz and a RAM of 16.0 GB performs the simulation analysis. To conduct the experiment, we developed nano drones with various sensors and transducers attached to their periphery (Figure 5), following the specifications listed in Table 2.

### 4.1. Simulation Analysis

**Figure 6:**
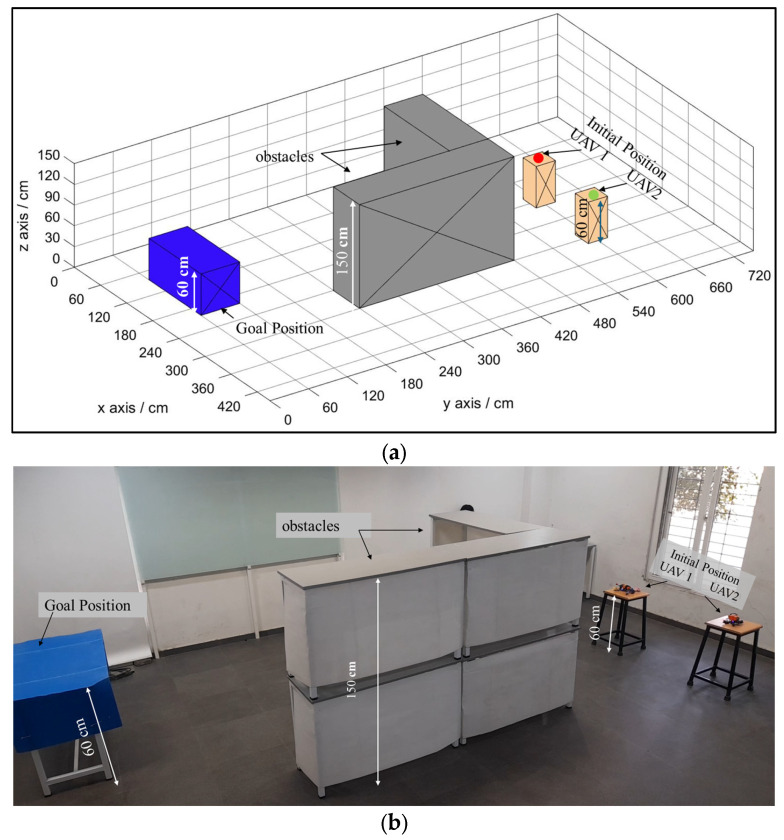
Environment containing start, obstacle, and goal. (**a**) Simulation environment created in MATLAB. (**b**) Real-time experimental environment created using stools and tables.

Simulation analysis is pivotal in the advancement and authentication of autonomous systems, particularly nano drones, particularly within intricate environments like indoor spaces containing both static and dynamic obstacles. Simulations furnish a virtual platform for the examination and enhancement of algorithms, presenting a cost-efficient and secure substitute for real-world experimentation. For simulation, we used MATLAB software, which is widely used and accepted by researchers due to its robust interface and capability for simulating robotic systems. The selection of this software was based on its appropriateness for modeling indoor environments and its capability to realistically simulate sensor data. The simulated indoor environment employed in our analysis is crafted to closely mimic real-world scenarios encountered by nano drones, as shown in Figure 6. The entire available floor space of 420 cm×720 cm×150 cm is divided into grids of 60 cm each. The initial positions of the drones are represented by yellow boxes measuring 30 cm×30 cm×60 cm; the goal position is represented by a blue box measuring 60 cm×30 cm×60 cm; and the obstacles are represented by fully occupied gray boxes with a maximum height of 150 cm. The boundary of the system is set to be 420 cm×720 cm×150 cm, which restricts the motion of drones within the mentioned range.

For developing an efficient path planning algorithm using NDA, the selection of the proper parameters is a crucial step. Separation weight (s), alignment weight (a), cohesion weight (c), food factor (f), enemy factor (e), velocity, and heading angle are a few important parameters that govern the performance of a drone. The range of these parameters is considered between 0 and 1 as shown in Table 1. To reduce the computation effort, increase the convergence speed, and avoid trapping into local minima, the number of search agents (dragonflies) is considered to be 60, and the maximum number of iterations is taken as 500. Based on the series of trials, the parameter values are selected, such as s=0.1, a=0.1, c=0.7, f=1, and e=1. During simulation, the drone is considered a point mass with an extended arm configuration. Its total mass is assumed to be 250 g, accounting for components such as four motors, propellers, a battery pack, and a main board. Each arm is set to a length of 0.036 m. The maximum traveling velocity of the drone towards the goal is considered as 0.1 m/s, and the maximum angle of turn for the drone is 40°.

**Figure 7:**
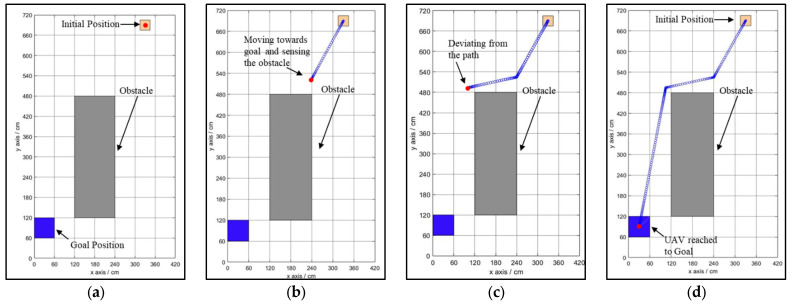
Path planning of a single drone in the presence of a static obstacle. (**a**) Drone at the initial point. (**b**) Drone moving towards the obstacle following the Euclidean distance. (**c**) Obstacle detected and deviation from the path. (**d**) Drone reaching the goal successfully.

**Figure 8:**
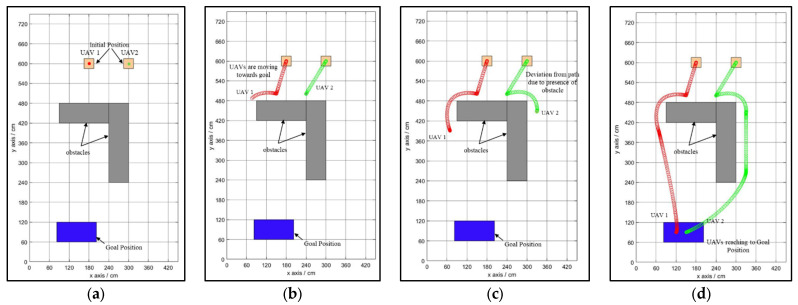
Path planning of multiple drones in the presence of a static obstacle. (**a**) Drones at the initial point. (**b**) Drones moving towards the obstacle following the Euclidean distance. (**c**) Obstacle detected and deviation from the path. (**d**) Drones reaching the goal successfully.

**Figure 9:**
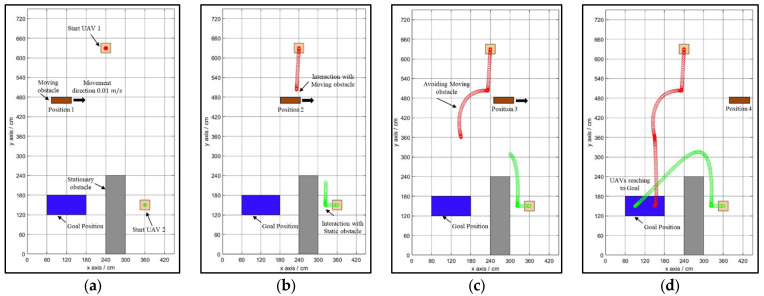
Path planning of multiple drones in the presence of a combination of static and moving obstacles. (**a**) Drones at the initial point. (**b**) Drones moving towards the obstacle following the Euclidean distance. (**c**) Drone 1 avoiding the moving obstacle and Drone 2 avoiding the static obstacle and deviating from the path. (**d**) Drones reaching the goal successfully.

The newly developed NDA approach is tested for a variety of environments, ranging from simple to complex. The controller based on the NDA approach can avoid the obstacle and reach the goal from the start position while selecting the optimal and smooth path in the case of a single drone, as shown in Figure 7a–d. The initial and goal positions are predefined by the drone. The drone starts its motion towards the goal in the direction of the shortest Euclidean distance shown in Figure 7b. Here, the drone detects the obstacle (an occupied cell) from a 20 to 60 cm margin, which forces the drone to deviate from the path to select the modified path to reach the goal (Figure 7c). At this position, the drone finds a clear, obstacle-free path to reach the goal (Figure 7d). The same controller is also tested for complex environments with multiple drones flying together in the environment with multiple obstacles, as shown in Figure 8a–d. In the case of multiple drones flying together, they have coordinated with each other to know the position of the obstacle and goal. They treat each other as obstacles to avoid collisions between them. Path planning in the presence of a moving obstacle, i.e., a dynamic environment, is a complex task to perform because the drone needs to check for the obstacle in real time and decide its new position as per the movement of the obstacle. The proposed NDA technique is successfully implemented for multi-drone environments with moving obstacles, as shown in Figure 9a–d.

The neighborhood dragonfly algorithm (NDA) improves the path planning for the drones by adding several improvements compared to existing algorithms such as the Artificial Potential Field (APF). At first, NDA utilizes a confined search approach, which allows each UAV to adapt its trajectory by considering adjacent UAVs instead of conducting a comprehensive global search. This method reduces the duration of computing work and accelerates the search procedure. In addition, NDA implements gradual changes to routes and facilitates concurrent processing, hence reducing the time required for planning. The system also includes adaptive convergence criteria, which allow for early termination when the best pathways are found, along with an efficient updating mechanism that maintains a balance between exploration and exploitation. As the outcome, there is a reduction in the average number of iterations. These features allow the NDA to effectively navigate around both stationary as well as moving obstacles in real-time, improving its adaptability and resilience for intricate UAV path planning assignments.

**Figure 10:**
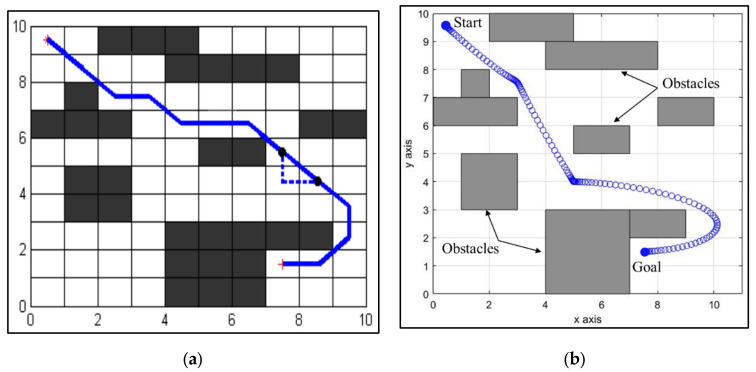
Comparison of path planning with IACO. (**a**) Path traced by the IACO in a static environment [39]. (**b**) Path traced by the NDA approach in a similar environment.

**Figure 11:**
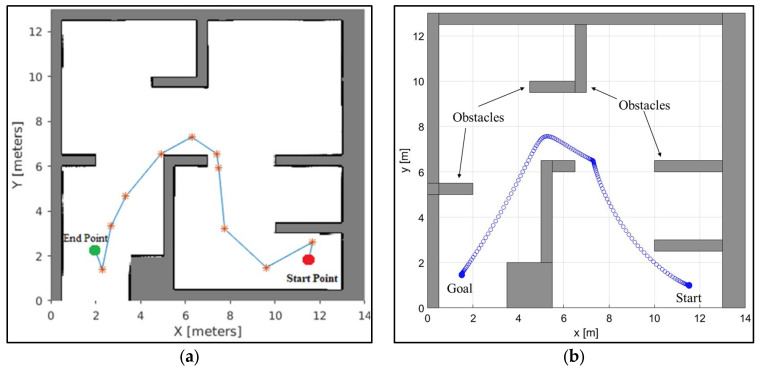
Comparison of path planning with PRM. (**a**) Path traced by PRM in a static environment [40]. (**b**) Path traced by the NDA approach in a similar environment.

We compare the proposed NDA approach with other existing AI techniques to verify its performance. At first, the proposed NDA approach is compared with intelligent ant colony optimization (IACO) for the simple environment of a 10 × 10 uniform grid. Comparison is performed by creating an environment of exactly the same size with the same obstacle position. It can be observed from Figure 10 that the path planned by the proposed technique is shorter and smoother. The percentage saved for the path length is 4.23%. The proposed NDA technique is also compared with the Probabilistic Roadmap (PRM) approach for trap-like environments, as shown in Figure 11. Here, the percentage path length saving is 5.41%. The comparison of simulation path length between the proposed NDA and other developed AI techniques can be seen in Table 3.

### 4.2. Experimental Analysis

For validating the results obtained from simulation analysis, real-world experimentation is performed in an indoor environment. By conducting experiments in a physical environment, we can evaluate the practical applicability and efficacy of the proposed NDA path planning algorithms framed for nano drones in indoor settings. This experimental validation is important for guaranteeing the reliability and resilience of our proposed approach. The experimental setup includes a controlled indoor environment made to replicate typical indoor settings generally faced by nano drones. The test area measures approximately 420 cm × 720 cm and includes a variety of static and dynamic obstacles to create a realistic scenario for path planning. For the experimentation, we built nano drones equipped with all necessary components for autonomous navigation, as shown in Figure 5. The drone is provided with a lightweight carbon fiber frame, high-speed brushless DC motors, and specialized propellers designed for optimal indoor flight. A flight controller powered by a Betaflight F4 flight controller served as the drone’s central processing unit, regulating motor output and flight dynamics. To enable real-time perception and obstacle detection in all directions, the drone integrated various sensors, such as LiDAR, infrared sensors, and a telemetric sensor. The detailed specification of drones is available in Table 2. The entire experiment has been conducted in indoor environments only, where the GPS module does not work properly. Thus, for the localization of drones within the environment, the Inertial Navigation System (INS) is adopted. Sensors like an accelerometer and a gyroscope are used to estimate the position and orientation of the drone within the environment. Positions of the start, goal, obstacles, and drones are recorded from side and top view cameras.

**Figure 12:**
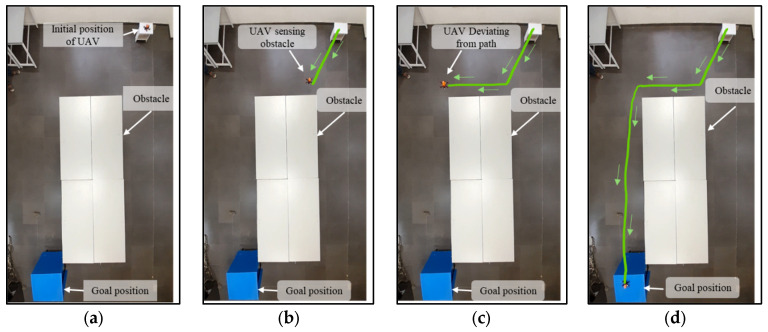
Experimental results of path planning of single drone in the presence of a static obstacle. (**a**) Drone at the initial point. (**b**) Drone moving towards the obstacle following the Euclidean distance. (**c**) Obstacle detected and the drone deviating from the path. (**d**) Drone reaching the goal successfully.

**Figure 13:**
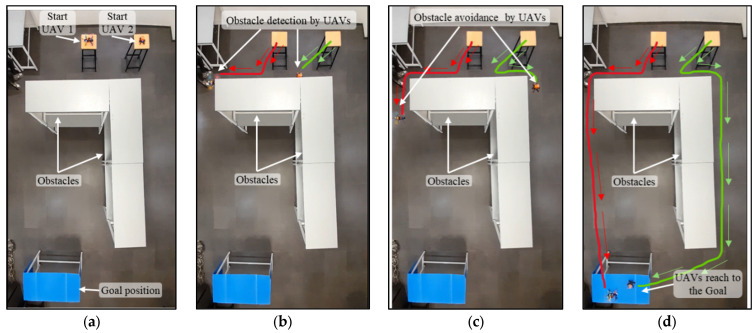
Experimental results of path planning of multiple drones in the presence of a static obstacle. (**a**) Drones at the initial point. (**b**) Drones moving towards the obstacle following the Euclidean distance. (**c**) Obstacle detected and the drones deviating from the path. (**d**) Drones reaching the goal successfully.

**Figure 14:**
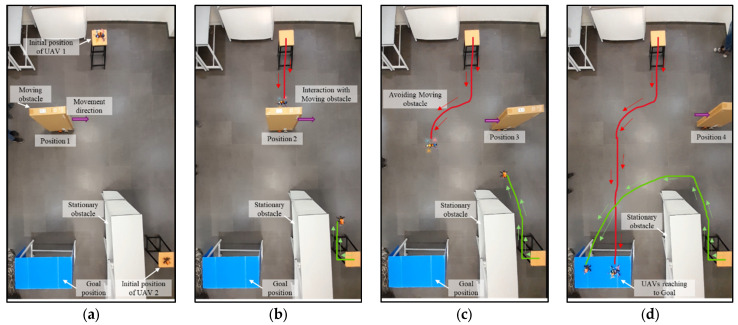
Experimental results of path planning of multiple drones in the presence of a combination of static and moving obstacles. (**a**) Drones at the initial point. (**b**) Drones moving towards the obstacle following the Euclidean distance. (**c**) Drone 1 avoiding the moving obstacle and Drone 2 avoiding the static obstacle and deviating from the path. (**d**) Drones reaching the goal successfully.

During the experiment, the initial position of the drone and the goal position are fixed. Obstacles are arranged either statically or dynamically, or in both manners. From the starting position, the drone starts to fly in the goal direction until it encounters the obstacle shown in Figure 12b. At this point, the proposed NDA-based controller activates and takes the decision to move the drone towards the obstacle-free goal path (Figure 12c). From this point, the drone achieves its maximum speed as there is no obstacle present and reaches the goal (Figure 12d). The experiment is also performed for multiple drones flying in the same environment, as shown in Figure 13a–d. Here, Drone 1 (shown by the red path) reached the goal faster as it was closer to it. The environment in Scenario 3 contains static as well as moving obstacles. An autonomous mobile robot has been programmed to move to-and-fro between locations (60,420) and (420,420) with 0.01 m/s velocity, as shown in Figure 14a–d. In this scenario, Drone 1 interacts with the moving obstacle and avoids it, while Drone 2 has a clear path with only one static obstacle. It can be seen clearly from Table 4 and Table 5 that in case of interaction with moving obstacle more time is required by the drone to reach to the goal compare to other scenarios though the path length is in similar range In the third scenario, the average time required to reach the target is 90.89 s, which is much higher compared to Drone 2, which is only 56 s.

### 4.3. Simulation vs. Experimentation Results

**Figure 15:**
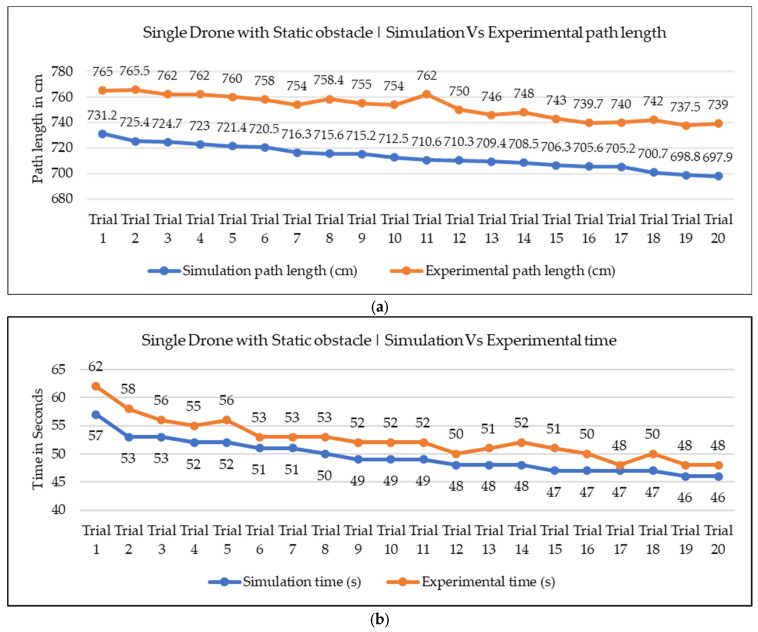
Simulation vs. experimental results comparison for single drone with a static obstacle: (**a**) path length in cm comparison; (**b**) comparison of required navigational time.

**Figure 16:**
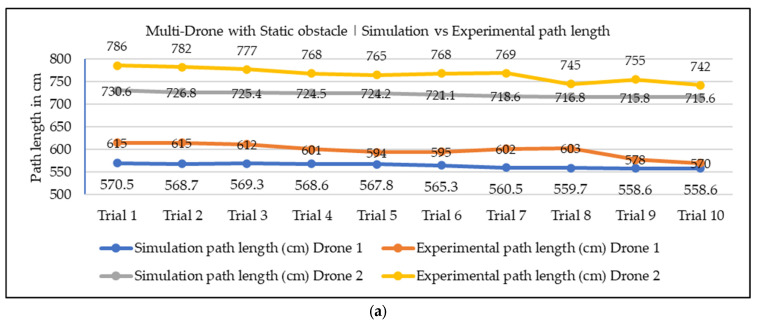

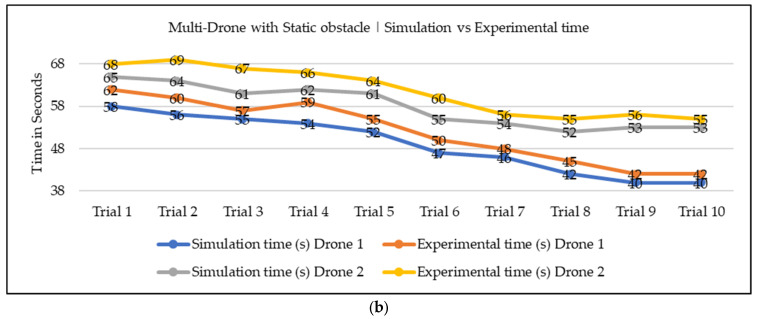
Simulation vs. experimental results comparison for multiple drones with a static obstacle: (**a**) path length in cm; (**b**) required navigational time.

**Figure 17:**
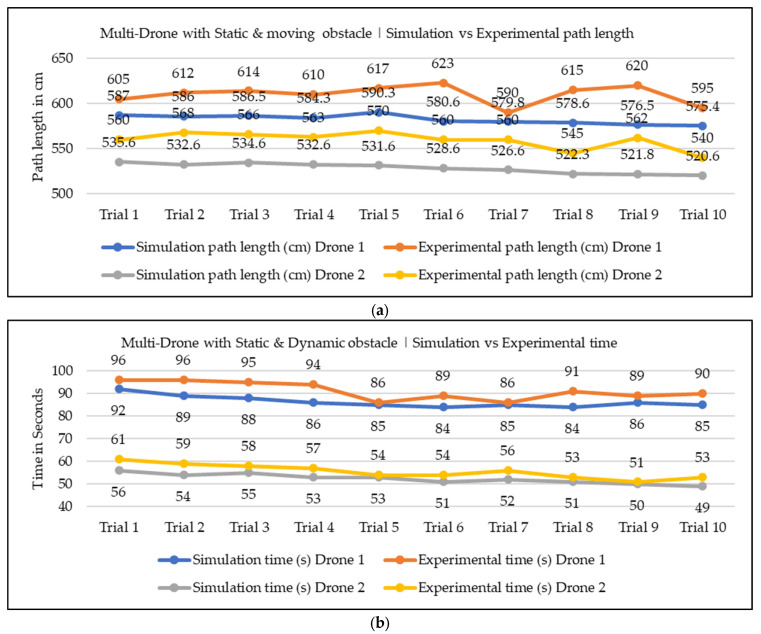
Simulation vs. experimental results comparison for multiple drones in a combination of static and dynamic obstacles: (**a**) path length in cm; (**b**) required navigational time.

In our current work, we conducted both simulation and experimentation analysis to assess the effectiveness of the proposed NDA approach for the path planning of nano drones in indoor environments. The simulation environment was aimed at building exactly the same environment as a real-world condition, considering factors like the start, goal, and obstacle positions, the dimensions of the environment, and the drone. However, it is important to note that some environmental variables, like wind velocity, drag resistance from air, and electrical resistance of drones, were not fully accounted for in the simulation study. Because of this, some errors were present in the simulation and experimental values. We focused our analysis on the two most crucial parameters of path planning, i.e., path length and time required for navigation. We conducted 20 trials for a single drone with static obstacle conditions, and 10 trials for multiple drones with static and moving obstacle conditions. The comparison graphs across all trials for path length and navigation time are presented in Figure 15, Figure 16 and Figure 17. Upon reviewing the comparison graphs, we can observe that the simulation results closely mirror the trends observed in experimentation, which indicates the effectiveness of the proposed NDA approach in all three scenarios. However, certain trials exhibited notable discrepancies, particularly in terms of path length. To further evaluate the consistency between simulation and experimentation results, we have taken the mean of all the trails, which is presented in Table 4 and Table 5. This analysis revealed that the obtained results from both simulation and experimental results are almost the same, and the percentage of deviation is less than 5.7% in all the scenarios. There are several factors associated with the small discrepancy in the simulation and experimental results. One potential factor is the assumptions and simplifications made in the simulation analysis, which do not fully capture the real-world scenarios. Adding to that, the environmental conditions and the accuracy of the sensors during the experiment could also contribute to the observed differences.

### 4.4. Comparison with Other Path Planning Algorithms

**Figure 18:**
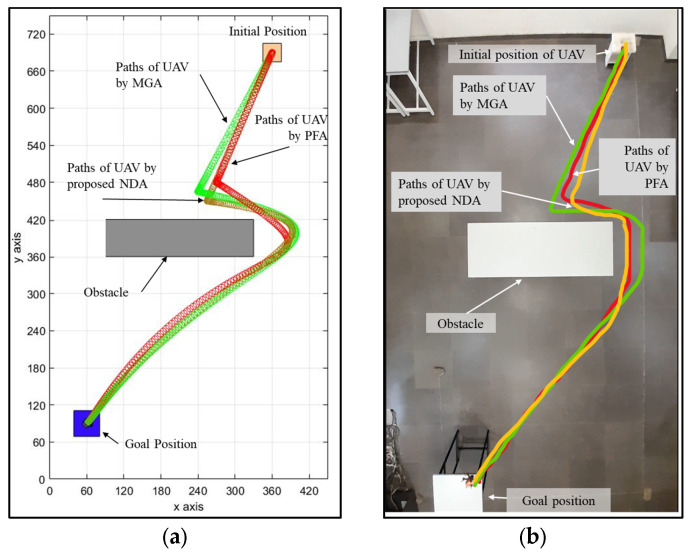
The path planning of UAV under a static environment by the MGA, PFL and NDA approach. (**a**) Simulation path by 3 approaches. (**b**) Experimental paths by 3 approaches.

To compare the performance of the neighborhood dragonfly algorithm (NDA), we compared it in detail with two well-known UAV path planning algorithms, namely the Matrix-based Genetic Algorithm (MGA) and Probability Fuzzy Logic (PFL). We conducted these comparisons under uniform conditions to provide an equitable evaluation of each algorithm’s performance across a range of scenarios. Figure 18a,b show the simulation experimental results of path planning under the static environment condition using three approaches. The comparison results from Table 6 demonstrate that the NDA achieves an average path length of 848.68 cm and 887.30 cm in simulation and experimentation, respectively, which is greatly shorter compared to MGA and PFL. The NDA’s localized interaction model helps it to discover more direct and efficient routes; at the same time, the MGA’s global search tends to produce slightly longer paths due to its iterative nature. PFL, with its preference for safer routes, typically generates the longest paths among the three. In terms of time travel, as shown in Table 7, the NDA once again demonstrated superior performance, with an average travel time of 76.36 and 80.00 s in simulation and experimentation, respectively. The MGA required a little bit more time, as it needed multiple iterations to refine the path. Meanwhile, PFL, which focuses on safety and meticulous obstacle avoidance, had the highest average travel time required. These results indicate NDA’s effectiveness in completing paths more rapidly, making it advantageous for real-time UAV operations.

## 5. Conclusions

The current work is focused on developing a novel path planning algorithm based on the neighborhood approach in the dragonfly algorithm, named the neighborhood dragonfly algorithm (NDA). The proposed NDA approach is implemented for the path planning application of the nano drone in indoor environments for a single drone with static obstacles, multiple drones with static obstacles, and multiple drones with static and moving obstacles. The collective behavior of dragonflies helps the NDA to facilitate localized interactions to facilitate effective navigation and obstacle avoidance in both static and dynamic environments. It is proven to be great at finding the optimal paths for drones while also making sure they work together and avoid collisions with each other. Importantly, our analysis shows that the difference in path lengths and the required navigational time between simulation and experimentation is minimal, with a variance of less than 5.7%. This shows the strong relationship between the simulation and experimental values, which justifies the robustness, reliability, and practical use of the proposed NDA approach. In comparison with other developed algorithms like IACO and PRM, the proposed NDA approach shows a better performance in terms of smooth path planning and path length optimization. The percentage savings in path length is more than 5% when compared to a similar environment. The results also demonstrate NDA’s significant superiority over MGA and PFL across key metrics such as path length and travel time. The path length traveled and required travel time by the drones using NDA are much lower compared to traditional algorithms. NDA presents a convincing and robust solution for UAV path planning, surpassing traditional algorithms by offering efficient, reliable, and scalable path planning capabilities. Its adaptability to environmental changes and adept navigation through static and dynamic obstacles make it an asset for UAV operations.

## Figures and Tables

**Table 1:** Parameters used in the DA algorithm.

S. No	Parameters	Value
1.	Number of search agents Nd	40–60
2.	Maximum number of iterations	500
3.	Separation weight s	0–1
4.	Alignment weight a	0–1
5.	Cohesion weight c	0–1
6.	Food factor f (target)	0–1
7.	Enemy factor e (obstacle)	0–1
8.	Lower bound of variable n (lb)	0
9.	Upper bound of variable n (ub)	10
10.	Number of variables (dim)	10
11.	Maximum time	600 s

**Table 2:** List of basic components that is used to build the drones.

S. No	Parameters	Value
1.	Frame	Carbon fiber frame
2.	Sensors	HC-SR04 ultrasonic sensor with a range of 2–400 cm, Sharp GP2Y0A41SK0F analog infrared distance sensor with a range of 4–30 cm, Benewake TFmini-S LIDAR sensor with a range of 0.1–12 m, optical flow sensor
3.	Flight Controller	Betaflight F4 flight controller with integrated gyro and accelerometer
4.	Motors	1104 brushless motors with a KV rating suitable for 2–3-inch propellers
5.	Propellers	Gemfan 2035 4-blade propellers for a good balance of thrust and efficiency
6.	Battery	2S (7.4 V) 450 mAh LiPo battery with a discharge rate of 45C
7.	IMU	MPU6050 IMU module for stable flight control
8.	Microcontroller	Arduino Nano for interfacing with sensors and implementing custom control algorithms
9.	Communication Module	NRF24L01+ 2.4 GHz wireless transceiver module
10.	Barometer	BMP280 barometric pressure sensor for altitude control
11.	Frame	Carbon fiber frame

**Table 3:** Comparison of path length of the NDA approach vs. other developed AI techniques.

Sl. No.	Simulation Path Length by Other Developed Techniques	Simulation Path Length by Proposed NDA Approach	% of Path Length Saved by NDA
Scenario-1 (Figure 10)	14.20	13.60	4.23%
Scenario-2 (Figure 11)	16.36	15.48	5.41%

**Table 4:** Simulation and experimental path length (cm) for single and multiple drones.

Sl. No.	Configuration	Drone	Average Simulation Path Length (cm)	Average Experimental Path Length (cm)	% Deviation
Scenario 1	Single drone with a static obstacle	Drone 1	712.96 (Figure 7)	752.06 (Figure 12)	5.20%
Scenario 2	Multiple drones with static obstacles	Drone 1	564.33 (Figure 8)	598.50 (Figure 13)	5.60%
		Drone 2	721.66	765.70	5.69%
Scenario 3	Multiple drones with static and moving obstacles	Drone 1	582.30 (Figure 9)	610.10 (Figure 14)	4.50%
		Drone 2	528.26	559.40	5.48%

**Table 5:** Simulation and experimental time (s) required to reach the goal from the start for single and multiple drones.

Sl. No.	Configuration	Drone	Average Simulation Time (s)	Average Experimental Time (s)	% Deviation
Scenario 1	Single drone with a static obstacle	Drone 1	49.50 (Figure 7)	52.50 (Figure 12)	5.65%
Scenario 2	Multiple drones with static obstacles	Drone 1	48.44 (Figure 8)	51.22 (Figure 13)	5.69%
		Drone 2	57.56	61.11	5.77%
Scenario 3	Multiple drones with static and moving obstacles	Drone 1	86.44 (Figure 9)	90.89 (Figure 14)	5.19%
		Drone 2	52.33	55.74	5.67%

**Table 6:** Simulation and experimental path length (cm) for a single drone with the MGA, PFL, and NDA approaches.

Sl. No.	Algorithm	Simulation Path Length (cm)	Experimental Path Length (cm)	% Deviation
1.	Matrix-based Genetic Algorithm (MGA)	861.60	901.00	4.37%
2.	Probability Fuzzy Logic (PFL)	867.65	915.35	5.21%
3.	**Neighborhood Dragonfly Algorithm (NDA)**	**848.68**	**887.30**	**4.35%**

**Table 7:** Simulation and experimental travel time for a single drone with the MGA, PFL, and NDA approaches.

Sl. No.	Algorithm	Simulation Path Length (cm)	Experimental Path Length (cm)	% Deviation
1.	Matrix-based Genetic Algorithm (MGA)	84.00	90.00	6.67%
2.	Probability Fuzzy Logic (PFL)	86.24	91.00	5.23%
3.	**Neighborhood Dragonfly Algorithm (NDA)**	**76.36**	**80.00**	**4.55%**

## Data Availability

Data is unavailable due to privacy restrictions.
